# Robust Fluorometric Aptamer Assay for Direct and Rapid Detection of Clinical Isolates of *Candida* spec.

**DOI:** 10.3390/ijms25063444

**Published:** 2024-03-19

**Authors:** Yiting Zhang, Hu Xing, Grigory Bolotnikov, Markus Krämer, Anil Bozdogan, Ann-Kathrin Kissmann, Tanja Weil, Barbara Spellerberg, Steffen Stenger, Frank Rosenau

**Affiliations:** 1 Institute of Pharmaceutical Biotechnology, Ulm University, Albert-Einstein-Allee 11, 89081 Ulm, Germany; yiting.zhang@uni-ulm.de (Y.Z.); hu.xing@uni-ulm.de (H.X.); grigory.bolotnikov@uni-ulm.de (G.B.); markus-1.kraemer@uni-ulm.de (M.K.); 2 Department of Laboratory Medicine, Medical University of Vienna, 1020 Vienna, Austria; anil.bozdogan@meduniwien.ac.at; 3 Max-Planck-Institute for Polymer Research Mainz, Ackermannweg 10, 55128 Mainz, Germany; weil@mpip-mainz.mpg.de; 4 Institute of Medical Microbiology and Hygiene, University Clinic of Ulm, Frauensteige 12, 89075 Ulm, Germany; barbara.spellerberg@uniklinik-ulm.de (B.S.); steffen.stenger@uniklinik-ulm.de (S.S.)

**Keywords:** *Candida*, biosensor, DNA aptamer, in vitro diagnostics

## Abstract

Infections caused by yeasts of the genus *Candida* are likely to occur not only in immunocompromised patients but also in healthy individuals, leading to infections of the gastrointestinal tract, urinary tract, and respiratory tract. Due to the rapid increase in the frequency of reported Candidiasis cases in recent years, diagnostic research has become the subject of many studies, and therefore, we developed a polyclonal aptamer library-based fluorometric assay with high specificity and affinity towards *Candida* spec. to quantify the pathogens in clinical samples with high sensitivity. We recently obtained the specific aptamer library R10, which explicitly recognized *Candida* and evolved it by mimicking an early skin infection model caused by *Candida* using the FluCell-SELEX system. In the follow-up study presented here, we demonstrate that the aptamer library R10-based bioassay specifically recognizes invasive clinical *Candida* isolates, including not only *C. albicans* but also strains like *C. tropcialis, C. krusei*, or *C. glabrata.* The next-generation fluorometric bioassay presented here can reliably and easily detect an early *Candida* infection and could be used for further clinical research or could even be developed into a full in vitro diagnostic tool.

## 1. Introduction

More than 20 species of the genus *Candida* are known to cause infections, with *Candida albicans* being the most prominent [1,2,3]. In the last three decades, *candidiasis* has emerged as the fourth most common case of blood infections in hospitals, due to the widespread use of antibiotics that destroy the competing bacterial flora and the prolonged systemic immunosuppressive treatments after organ transplantation and chemotherapy [4,5,6,7,8]. Other factors that predispose to candidiasis are pregnancy or the use of hormonal contraceptives that reduce the acidity of the vaginal environment and can lead to Vulvovaginitis candidomycetica, which leads to an annual loss of productivity of up to USD 14–39 billion in high-income countries [9,10]. Furthermore, invasive candidiasis is the cause of more than 250,000 infections and 50,000 deaths worldwide [11,12,13,14,15,16]. This makes *Candida*’s timely and accurate detection crucial for diagnosis and the subsequent treatment. The conventional diagnostic approach is to accurately identify *Candida* spp. in clinical samples based on morphological and physiological characteristics, which is complex and can be time-consuming; however, more rapid commercial systems may also inevitably face serious sensitivity problems [17,18]. Newer detection methods, such as matrix-assisted laser desorption/ionization time-of-flight mass spectrometry (MALDI-ToF), may also be impractical for many laboratories in developing countries as such complex methods mostly rely on pure cultures, and they are not only highly expensive but also very time-consuming [19,20,21,22,23]. Other standard detection methods based on quantitative PCR (qPCR) have their drawbacks due to their restricted sensitivity and limited specificity for only a rather small and pre-defined set of *Candida* spec. [24,25,26]. Therefore, novel techniques that can identify *Candida* easily, quickly, and successfully with respect to the economic aspects appear to be of particular importance.

In the last three decades, aptamers have emerged as new ligands that not only have similar properties to antibodies but are more stable and easier to synthesize and modify and have higher affinity towards their dedicated targets [27,28,29,30,31,32,33,34]. Therefore, aptamers can be used as ligands instead of antibodies to detect pathogens like bacteria or fungi [35,36]. Fluorometric aptamer-mediated whole-cell detection methods are recognized as a promising tool for the development of fast, specific, and clinically applicable bioassays [37,38]. In fact, functional aptamer-based assays have been introduced against a variety of health-relevant target organisms, including major pathogens [39,40,41,42,43,44,45,46,47], as well as for potential probiotic human gut bacteria [48]. However, what most of these approaches have in common is that they require additional technical components like different types of (nano)particles or they rely on sandwich-type assay principles [49]. In contrast, more simple assays have been suggested; these were based on the direct labeling of the intended target with enriched (also known as polyclonal) aptamer libraries or individual aptamers without the need for secondary binding molecules or enzyme-mediated signal amplification [47,48,49,50,51,52,53].

With *C. albicans*, *C. auris*, and *C. parapsilosis* as target cells for a whole-cell SELEX process (Systematic Evolution of Ligands by EXponential enrichment) [32,33], we developed an enriched aptamer library against this class of important human pathogens in a previous study [50]. This library was already sufficient to label these *Candida* species with fluorescence and allowed fungal cells to be distinguished from human dermal fibroblast (HDF) cells via fluorescence microscopy in a skin early infection model [50]. However, the intensity of the unspecific background signals obtained with the HDF cells in a fluorometric suspension assay suggested a considerable potential to improve the sensitivity of such an enriched SELEX library while simultaneously enhancing the specificity for *Candida* spec. and improving their differentiation from human cells. Thus, additional rounds of SELEX were performed, again with a mixture of the three *Candida* spec. and counter selection using human fibroblasts. The intended improvements were verified using not only HDF cells but also a set of three additional lines of somatic cells, including colorectal adenocarcinoma cells (HT29), pancreatic cancer cells (MIA-PaCa-2P), and breast cancer cells (MCF7). Enriched aptamer libraries of this type, in their fluorescently labeled versions, offer an efficient option with which to label target cells with high specificity, allowing sensitive measurements simply by using fluorometric suspension assays. Without the need for additional assay components or signal amplification measures, it was possible to distinguish a set of 87 clinical isolates from invasive infections with different *Candida* spec. from the human cells. This final library was also characterized by high affinities against the *Candida* spec., with dissociation constants in a low nanomolar range and a low detection limit. The sensitivity, but also the intriguing ease with which the cells of *Candida* species were efficiently distinguished from human cells in this suspension assay measured with the standard fluorometer equipment may open new avenues for clinical diagnostics of pathogens based on enriched libraries or selected individual aptamers labeled with fluorescent dyes. We believe that this *Candida* spec.-specific assay may represent a prototype assay, and we suggest the name Flu*Cand*A-Assay (“Fluorescence *Candida* Aptamer”) and hope to inspire other researchers to develop similar concepts, which could rapidly enlarge the portfolio of fast and reliable detection technologies for health-threatening organisms.

## 2. Results

**Figure 1:**
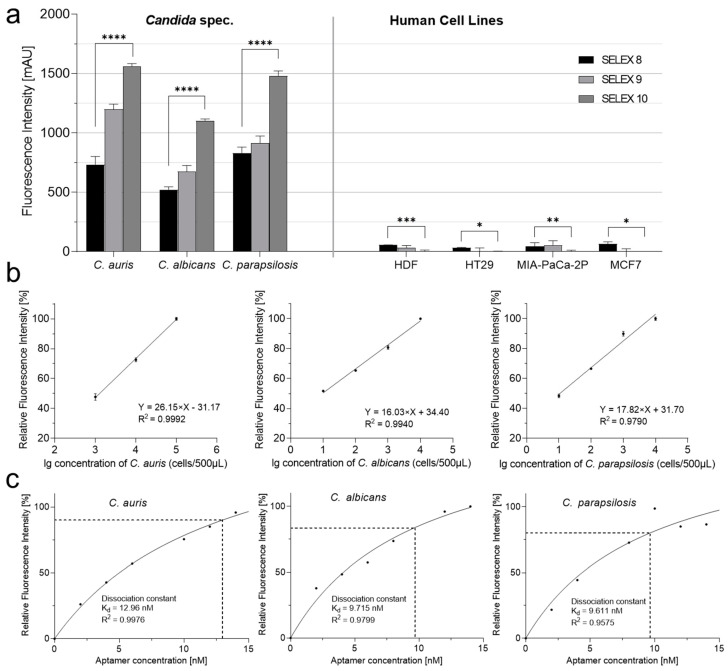
Increased aptamer affinities towards *Candida* spec. cells allows their differentiation from four human cell lines. (**a**) Based on the aptamer library R8 [50], two additional rounds of SELEX delivered libraries R9 and R10. Human cell lines to be differentiated from *C. auris*, *C. albicans*, and *C. parapsilosis* were HDF, HT29, MIA-PaCa-2P, and MCF7. Binding assays were performed using Cy5-labeled R8, R9, and R10 aptamer libraries. The fluorescence intensity can be correlated with the affinity of the aptamers against the given target. *p* values < 0.05 were considered significant. * *p* denotes < 0.05, ** denotes *p* < 0.01, *** denotes *p* < 0.001 and **** denotes *p* < 0.0001. (**b**) Determination of lower detection limits (black lines) of aptamer library R10. Relative fluorescence against logarithmically scaled cell numbers of *C. auris*, *C. albicans*, and *C. parapsilosis* with 10 pmol of Cy5-labeled aptamer. Linearly fitted plot represents lower detection limit X = lg(cell number), Y = (relative fluorescence intensity). (**c**) Determination of dissociation constants (K_d_ values) (black dotted lines) of the aptamer library R10 for *C. auris*, *C. albicans*, *C. parapsilosis* by determination of the percentage of bound aptamers (*y*-axis) against an increasing of aptamer concentration up to 15 pmol. By performing an exponential fit K_d_ values of 12.96 nM for *C. aurius*, 9.715 nM for *C. albicans*, and 9.611 for *C. parapsilosis* were computed. All experiments were conducted as triplicates (*n* = 3).

Based on the published enriched anti-*Candida* SELEX library R8 [50], two additional rounds of selection were performed to evaluate the resulting affinities against the *Candida* target strains and their specificities with HDF cells and three further cell lines as controls. As intended, both the affinity and the specificity increased significantly, with the affinity improving up to twofold in the resulting novel final library R10. In addition, R10 showed no binding and thus no fluorescent labeling of the human cell lines (Figure 1a). This allowed a lower detection limit (defined as 50% of the maximal observable fluorescence at the given amount of aptamers) of 2000 cells per milliliter for *C. auris*, but as low as 20 cells for both *C. albicans* and *C. parapsilosis*. Accordingly, the dissociation constants (K_d_ values) were found to be reasonably low and were in the nanomolar range from 10 to 13 nM, respectively. The curves could be fitted by a typical single site-specific binding model [51] and showed reasonably low dissociation constants (K_d_ values) of 12.96 nM, 9.715 nM, and 9.611 nM, respectively.

**Figure 2:**
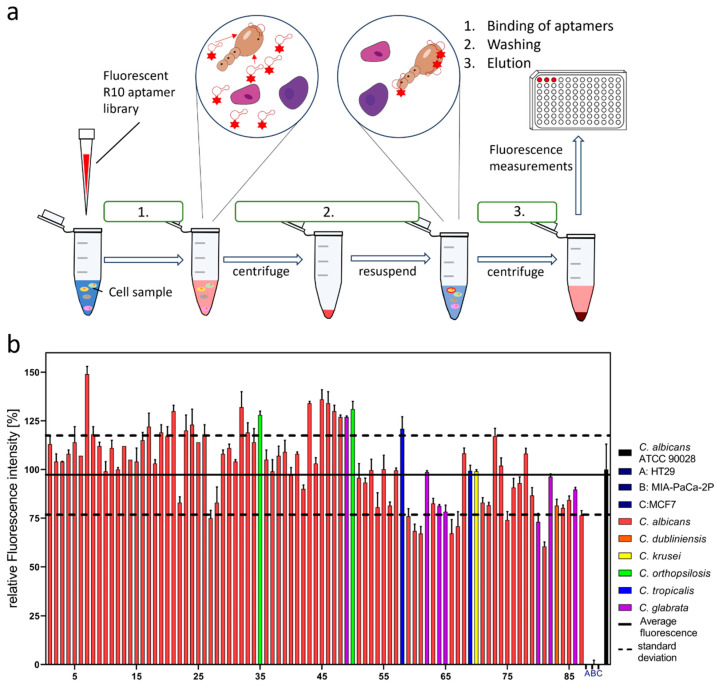
(**a**) Schematic of binding assay with Cy5 fluorescent-labeled R10 aptamer library. The fluorescent aptamers are first incubated with the target cells, centrifuged, and resuspended in PBS buffer. The resulting aptamer target complexes are then separated by denaturation at 95 °C for 5 min. The solution is centrifuged, and the fluorescence of the supernatant is measured. (**b**) Binding assay of aptamer library R10 against a multitude of clinical isolates of *Candida* (*n* = 87) of 6 different species, *C. albicans*, *C. dubliniensis*, *C. krusei*, *C. orthopsilosis*, *C. tropicalis*, *C. glabrata* (sorted by sampling number), were conducted, with A, B, C representing the human cell lines (HT29, MIA-PaCa-2P, MCF7). The laboratory strain *C. albicans* ATCC90028 was used as the control and represents a relative fluorescence of 100%. A detection window was found by determining the average relative fluorescence of the clinical isolates (solid black line) and calculating the 1 sigma interval (dotted black lines) 0.98 ± 0.21.

The sensitivity and specificity of the enriched R10 library suggested that our aim to develop an easy but reliable assay to distinguish *Candida* spec. from human cells might be achievable and realistic. The suggested assay follows the workflow demonstrated in Figure 2a and consists of three principal steps: (1) binding, (2) washing, and (3) elution, prior to the final fluorescence measurement involving only two subsequent centrifugation steps in the experiment (Figure 2a).

As a proof of concept, to show the principal functionality of this assay’s workflow, we intended to demonstrate that a considerably large ensemble of real clinical isolates of the genus *Candida* (*Candida* spec.) could be differentiated from the human control cells as desired. During a sampling campaign, 87 individual isolates were collected in the university hospital Ulm and were provided anonymously for this study (numbered according to the order of their date of sampling in the campaign, as given in Figure 2b). This ensemble harbored *C. albicans* as the predominant species, represented by 74 individual strains as well as 5 additional *Candida species*. Among these, with seven representatives, *C. glabrata* was the second most frequent species, followed by *C. orthopsilosis* and *C. tropicalis* with two isolates each, and *C. dublinensis* and *C. krusei* were each represented by only a single isolate. Interestingly, as expected for a larger German hospital, *C. auris*, which is probably the most relevant and health-threatening Candida pathogen emerging in various other regions of the world, was not present in the cohort of patients who delivered the 87 clinical samples. The assay was performed as described for the 87 clinical isolates and the laboratory strain *C. albicans* ATCC 90028 as a reference. Moreover, the target negative controls represented by the set of human cell lines were again also included, and it was found, as expected, that they delivered a zero-fluorescence signal. The fluorescence signal of *C. albicans* ATCC 90028 was judged to be the reference and was set to 100% labeling efficiency, and all the other measurements were normalized accordingly. The arithmetical mean was calculated for all the samples (except the human cell lines), and the positive and negative standard deviations were used to define a detection window (0.98 ± 0.21). The vast majority of isolates delivered fluorescence signals within or above the borders of the detection window, with only four individual *C. albicans* isolates falling slightly short of the lower border. Interestingly, the non-*albicans Candida* strains could also be confidently measured and thus distinguished from human cells.

## 3. Discussion

With the finding that polyclonal aptamer libraries originating directly from SELEX processes may outperform the individual aptamers [52] selected from these libraries, we introduced such enriched or “polyclonal” aptamer libraries as valuable tools for the fluorescent labeling of target structures and thus the specific quantification of a series of microbial symbionts and pathobionts [52,53,54,55,56,57]. Moreover, the functionality of this concept was further demonstrated with different tissues of plant roots [58] and by using specific enriched libraries for the construction of electronic gFET-based biosensors to measure pre-diabetes-related biomarkers [59]. The considerable general potential of oligonucleotide aptamers for the detection of pathogens and, in turn, for the development of assays for monitoring, and therefore disease control involving a variety of aptamer-based bioassays, including lateral flow assay concepts and colorimetric assays, as well as fluorescence-based concepts, is widely accepted and has recently been nicely reviewed by Wan and coworkers [60]. One key challenge in diagnostic assays for pathogen detection is to discriminate the dedicated harmful microbes in the sample preparations for analysis from “contaminating” human cells. We have recently shown that an enriched library against different ***C****andida* strains could already be used to distinguish the yeast cells from human cells in this case and was exemplified solely by human dermal fibroblasts (HDF), which served as so-called counterselection targets during the respective SELEX process [50]. It turned out that background detection of HDF cells and other human cell lines was low but significant, particularly in fluorometric binding assays, suggesting a remnant affinity for (experimentally yet undefined) epitopes on human cells. According to the dogma of directed evolution “you get what you screen for”; we decided to use the power of SELEX and to improve the specificity of the library by eliminating the vexatious background affinity with a few additional harsh rounds of selection and counterselection. Interestingly, in addition to this, the affinity of the final library for its dedicated target cells was also significantly enhanced, as measured by the fluorescence-labeling intensity of the different *Candida* strains. The affinity towards the reference *Candida* strains *C. albicans*, *C. auris*, and *C. parapsilosis* was in a reasonably low nanomolar range that was comparable to established diagnostic antibodies and aptamers [61,62,63,64,65,66]. This resulting high affinity-enriched aptamer library was used to test the Flu*Cand*A concept in a fast and easy assay for the detection of *Candida* spec. that could distinguish the *Candida* spec. from the human cells in the background. The strains from the set of clinical isolates were preponderantly encompassed positively by the assay, leaving only 4 of the 87 tested samples as false-negatives, but in these cases, the high fluorescence values lay just outside the detection window at the lower limit. We believe that this proof of the Flu*Cand*A concept may serve as the experimental basis with which to approach a feasible diagnostic assay for the further in-depth evaluation of its general potential to detect different *Candida* species. This evaluation should include larger cohorts of clinical isolates as well as larger ensembles of *Candida* reference strains. The same enriched aptamer library may also serve as a valuable pool of diverse sequences to isolate individual aptamers against specific *Candida* species or even strains. We hope that the portfolio of possible applications may also inspire and enable the development of assay systems of higher complexity, like the construction of electronic biosensors.

## 4. Materials and Methods

### 4.1. Cell Lines and Cell Culture

Experimental yeast strains, including *C. auris* (DSMZ-No. 21092), *C. albicans* (ATCC90028), *C. parapsilosis* (ATCC22019), and the clinic isolates, were inoculated in 5 mL of RPMI (Roswell Park Memorial Institute) medium (Thermo Fisher Scientific, Waltham, MA, USA) and cultured at 37 °C. All the clinical isolates of the *Candida* species were identified by matrix-assisted laser desorption ionization time-of-flight mass spectrometry (Maldi-ToF MS, Bruker Corporation, Billerica, MA, USA) and provided by patient samples sent to the microbiology department for diagnostic purposes. The strains were collected anonymously; thus, it was not possible to assign the strains to patients. The accreditation number of the microbiology department is DIN EN ISO15189:2014 (DAkks). The human cells, including HDF, HT29, MCF7, and MIA-PaCa-2P, were incubated in DMEM medium, 1% (*v/v*) Minimal Essential Medium Non-Essential Amino Acids, MEM NEAA(Life Technologies, Carlsbad, CA, USA), 1% (*v/v*) Penicillin/Streptavidin (Life Technologies, Carlsbad, CA, USA), 15% (*v/v*) Fetal Calf Serum (FCS) (Life Technologies, Carlsbad, CA, USA), and 83% (*v/v*) Dulbecco’s Modified Eagle Medium (DMEM) (Life Technologies, Carlsbad, CA, USA) and cultured in a 37 °C cell culture incubator containing 5% CO_2_.

### 4.2. Cell Pretreatment

After treatment with Accutase^®^ (Life Technologies, Carlsbad, CA, USA), 20,000 cells were removed, added to a 96-well plate, and incubated at 37 °C for 24 h in a cell incubator.

### 4.3. Cell SELEX

#### 4.3.1. Pretreatment before Counter SELEX

After being treated with Accutase^®^, the desired HDF cells were removed and added to 96-well plates (20,000 cells) or 24-well plates (100,000 cells), followed by incubation at 37 °C for 24 h in a cell culture incubator to re-adhere the cells. The cells were carefully washed once with 1× PBS after removing the medium before the screening.

#### 4.3.2. Pretreatment before Target SELEX

*C. auris*, *C. albicans*, and *C. parapsilosis* were centrifuged at 9000× *g* for 2 min and washed with 1× PBS.

#### 4.3.3. Aptamer Activation

Add the aptamer library to 500 µL of 1× PBS, incubate at 95 °C for 5 min, place in an ice bath for 5 min, and then leave for 20 min at room temperature.

#### 4.3.4. Screening

The screening process for rounds 1–8 can be found in the previous article [50]; this was followed by two additional rounds of screening, as shown in Table 1.

The activated library was incubated with adherent HDF cells at 37 °C for 1 h. The supernatant was then carefully aspirated; BSA (100 mg/mL) and tRNA (10 mg/mL) were added to increase stringency and incubated with *Candida* at 37 °C for 30 min, followed by centrifugation at 9000× *g* for 2 min, the removal of the supernatant, and the final washing with 1× PBS to remove unbound aptamer from the precipitate (see Table 1).

#### 4.3.5. Elution

The cells from the previous step were resuspended in 100 µL of 1× PBS and incubated at 95 °C for 5 min, followed by centrifugation at 11,000× *g* for 1 min to collect the *Candida*-bound aptamer.

#### 4.3.6. Library Amplification

PCR further amplified the aptamers collected in the previous step. The amplification conditions were as follows: 3 min at 95 °C, followed by 25 cycles of 30 s at 94 °C; 30 s at 56 °C; 10 s at 72 °C; and finally, 2 min at 72 °C. Next, the PCR products were purified (MACHEREY-NAGEL GmbH & Co. KG, Düren, Germany). The resulting double-stranded DNA was broken down into single-stranded DNA by λ-nucleic acid exonuclease catalysis (New England Biolabs, Ipswich, MA, USA) and finally purified by an optimized PCR purification kit (MACHEREY-NAGEL GmbH & Co. KG, Düren, Germany). The binding buffer required for this purification process was supplemented with 1.5 volumes of isopropanol and 10 µL of natrium acetate solution (pH 5) to increase the yield of single-stranded DNA.

#### 4.3.7. Binding Assay

##### *Candida* Binding Assay

After washing the yeast according to Section 4.3.2, 20,000 cells were used for analyses and incubated with 5 pmol of activated aptamer library in 500 µL of PBS for 30 min at 37 °C. Next, the culture was centrifuged at 9000× *g* for 2 min to remove the supernatant and washed once. The precipitate was resuspended in 100 µL of 1× PBS buffer to obtain the eluted cell junctional aptamers, and the fluorescence intensity was determined by measuring at an excitation wavelength of 637 nm and an emission wavelength of 670 nm using an Infinite M200 spectrophotometer (TECAN, Männedorf, Switzerland).

##### Cell Binding Assay

After re-culturing 20,000 individual cells in 24-well plates, the cells were incubated with 5 pmol of activated aptamer library in 500 µL of PBS at 37 °C for 30 min. The supernatant was removed and treated with 200 µL of accutase. After centrifugation for 3 min at 2000× *g*, the cells were washed once with 500 µL PBS and measured according to Section 4.4.

### 4.4. Affinity Analysis

The binding affinity of the selected aptamer libraries was determined by incubating 20,000 *C. auris*, *C. albicans*, and *C. parapsilosis* in 500 µL of PBS with different concentrations of the aptamers. Finally, the dissociation constants (Kd) of the aptamer libraries were determined by fitting the dependence of the fluorescence intensity on the aptamer concentration to the equation Y = B_max_ × X/(K_d_ + X) using GraphPad PRISM 8. (GraphPad Software, San Diego, CA, USA), with Y = the measured fluorescence, B_max_ = the maximal fluorescence, and X = concentration of the aptamers).

### 4.5. Sensitivity Test

The sensitivity of the aptamers was determined by analyzing the linear relationship between fluorescence intensity and the log value of the cell number for each yeast.

### 4.6. Detection of Clinic Isolates

One milliliter of clinic isolates with an OD of 0.01 was incubated with 5 pmol of aptamer library, and the fluorescence intensity of the *C. albicans* strains under the same conditions as above was compared to determine the ability of the aptamer library to detect *Candida* in practice.

## Figures and Tables

**Table 1:** SELEX procedure for rounds 9–10.

SELEX Rounds	Aptamer [pmol]	Counter SELEX with HDF	Target SELEX with *Candida*	Wash Times	BSA/tRNA [pmol]
9	0.1	20,000 cells	250 μL OD600 = 1	8	3000
10	0.1	100,000 cells	250 μL OD600 = 1	8	3000

## Data Availability

The data can be requested from the authors for valid reasons.
